# Magnetic Resonance Imaging of Patellofemoral Morphometry Reveals Age and Gender Variations in the Knees of Children and Adolescents

**DOI:** 10.3390/diagnostics11111985

**Published:** 2021-10-26

**Authors:** Wonchul Choi, Sang-June Lee, Jongbeom Oh, Hongseok Baek, Jinhyuk Yang, Jaeyeon Shin, Bosung Jung, Soonchul Lee

**Affiliations:** 1 Department of Orthopaedic Surgery, CHA Bundang Medical Center, CHA University School of Medicine, Pocheon 13496, Gyeonggi-do, Korea; wcosdoc@gmail.com (W.C.); jongbumoh@gmail.com (J.O.); 2 Department of Orthopaedic Surgery, Wonju Severance Christian Hospital, Yonsei University Wonju College of Medicine, Wonju 26426, Korea; lsjshock@gmail.com; 3 CHA Graduate School of Medicine, CHA University, Pocheon 13496, Gyeonggi-do, Korea; hongseokbaek@gmail.com (H.B.); jhyang499@gmail.com (J.Y.); greentea1bo@gmail.com (B.J.); 4 Department of Computer Science, College of IT Engineering, SeMyung University, Jechun 27136, Korea; zhyeon838@gmail.com

**Keywords:** patellar dislocation, patellar instability, trochlea dysplasia, patellofemoral joint

## Abstract

Background: The morphology and alignment of the patellofemoral joint are crucial risk factors for patellar instability, and the incidence of acute primary patellar dislocation is the highest in women in their second decade of life. The purpose of the study was to analyze age and gender variations of the patellofemoral joint using magnetic resonance imaging (MRI). Methods: A total of 852 patients aged between 4 and 18 years with a history of knee MRI examinations were screened for eligibility and 663 patients (470 males, 193 females) were included. Patients were divided into groups according to age and sex. The age group was divided into five groups (Group 1, 4–6 years; Group 2, 7–9 years; Group 3, 10–12 years; Group 4, 13–15 years; and Group 5, 16–18 years). Three orthopaedic surgeons measured MRI parameters reflecting the patellofemoral morphology (sulcus angle, lateral trochlear inclination, trochlear facet symmetry, and femoral depth) and alignment (tibial tuberosity–trochlear groove distance, percent sulcus location, and percent tibia tuberosity location). Results: Parameters including tibial tuberosity–trochlear groove distance, sulcus angle, percent tibial tuberosity location, trochlear facet symmetry, and femoral depth showed significant differences between the age groups (*p* < 0.05). The sulcus angle decreased fin Group 2, and the femoral depth showed an increasing trend with aging in male patients. However, the sulcus angle in females decreased first and then increased in Group 3 as the inflection point. The femoral depth showed an opposite pattern. Conclusions: Patellofemoral morphometry showed age and gender variation. Notably, the sulcus angle and femoral depth were significantly different between males and females and changed according to the development. These findings may reflect the sex difference and peak incidence of the patellar instability risk. Understanding the morphological changes and differences of the patellofemoral joint may facilitate the diagnosis of patellofemoral pathologies.

## 1. Introduction

The morphology and alignment of the patellofemoral joint, such as trochlear dysplasia, patella alta, and excessive lateralization of the tibial tuberosity, is a key risk factor contributing to patellar instability [1,2,3]. The incidence of acute primary patellar dislocation ranges from 23.2 to 77 per 100,000 population and is the highest among women in their second decade of life [4,5,6,7,8,9,10,11]. Among them, 69% of primary patellar dislocations were aged between 10 and 19 years, with a 1.61-fold higher incidence in women than in men [6,12].

Several studies reported morphological and anatomic differences in patellar dislocation of patients’ knees using magnetic resonance imaging (MRI) compared with a normal knee [13,14,15,16,17]. However, only a few studies have investigated the morphometric changes in the patellofemoral joint of the skeletally immature knee between males and females with aging [18,19,20]. Trivellas et al. suggested a study of trochlear morphology development in 246 normal knees via MRI, but this study involved a small sample size and a limited age group (between 3 and 16 years) [18]. In this study, only trochlear parameters (Trochlear height, Sulcus angle, and Trochlear slope) were analyzed. A retrospective study by Eric et al. revealed normative data of 17 parameters related to the knee joint. However, this study was conducted on Western people, and the sample size was too small (132 MRI) to standardize the normative data [19]. The study of cadaveric knees was limited to ages between 2 and 11 years using computed tomography (CT) [21]. Since this study entailed osseous measurement, it may differ from the actual patellofemoral articular surface relationship. Shital et al. performed a morphometry analysis of trochlear dysplasia in adolescence; however, these patients underwent reconstruction of the medial patellofemoral ligament (MPFL) [20]. This study was analyzed only for the trochlear parameters. Interpretation of MRI images of skeletally immature knees is often inaccurate due to continuous developmental changes and lack of standardization of normal parameters. It is also difficult to interpret MRI findings due to sex variation and differences in cartilage and bone characteristics of the knee joint. Understanding developmental changes in the normal skeletally immature knee according to sex is important to interpret the pathology of patellofemoral instability. It is essential to analyze the serial sex-related developmental changes of the patellofemoral joint in a large sample population. 

The purpose of the study was to analyze age and gender variations of the patellofemoral joint using MRI. We hypothesize that sex differences exist in the development of the patellofemoral joint of the skeletally immature knee. 

## 2. Materials and Methods

### 2.1. Study Population

**Figure 1:**
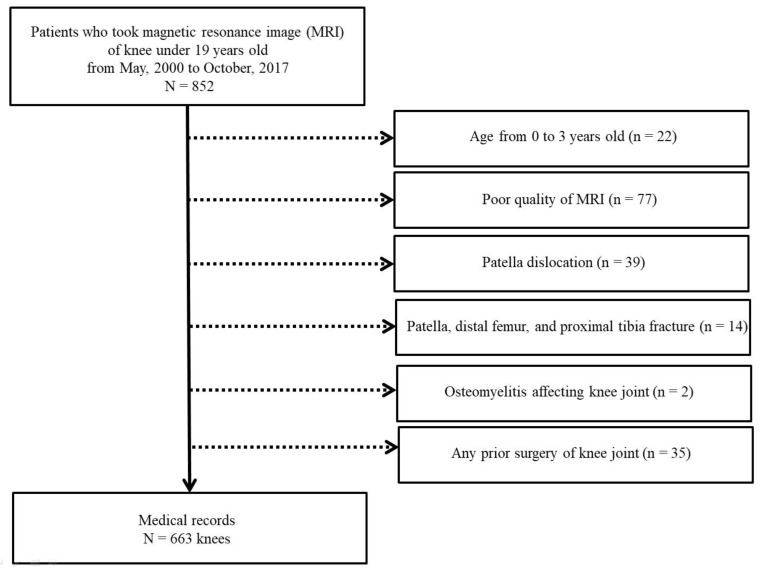
Flow chart showing the study population selection.

A total of 852 patients below 19 years of age who underwent an MRI of the knee joint from May 2000 to October 2017 were retrospectively reviewed. Normal knee MRI was selected using KCD-8 codes (Korean Standard Classification of Diseases) including knee contusion, knee laceration, knee pain, knee benign mass, meniscus tear, cellulitis, and knee sprain. Among them, 189 patients were excluded because of age less than three years, poor image quality, patellar dislocation, fractures involving patellofemoral joint, osteomyelitis affecting knee joint, and prior surgery of knee joint (Figure 1). The zero- to 3-year-old population was excluded due to the low sample size. Patients were divided into five groups according to age and sex (Group 1, 4–6 years; Group 2, 7–9 years; Group 3, 10–12 years; Group 4, 13–15 years; and Group 5, 16–18 years). Our institutional review board approved this study (CHAMC-2020-06-030).

### 2.2. Radiographic Measurement

**Figure 2:**
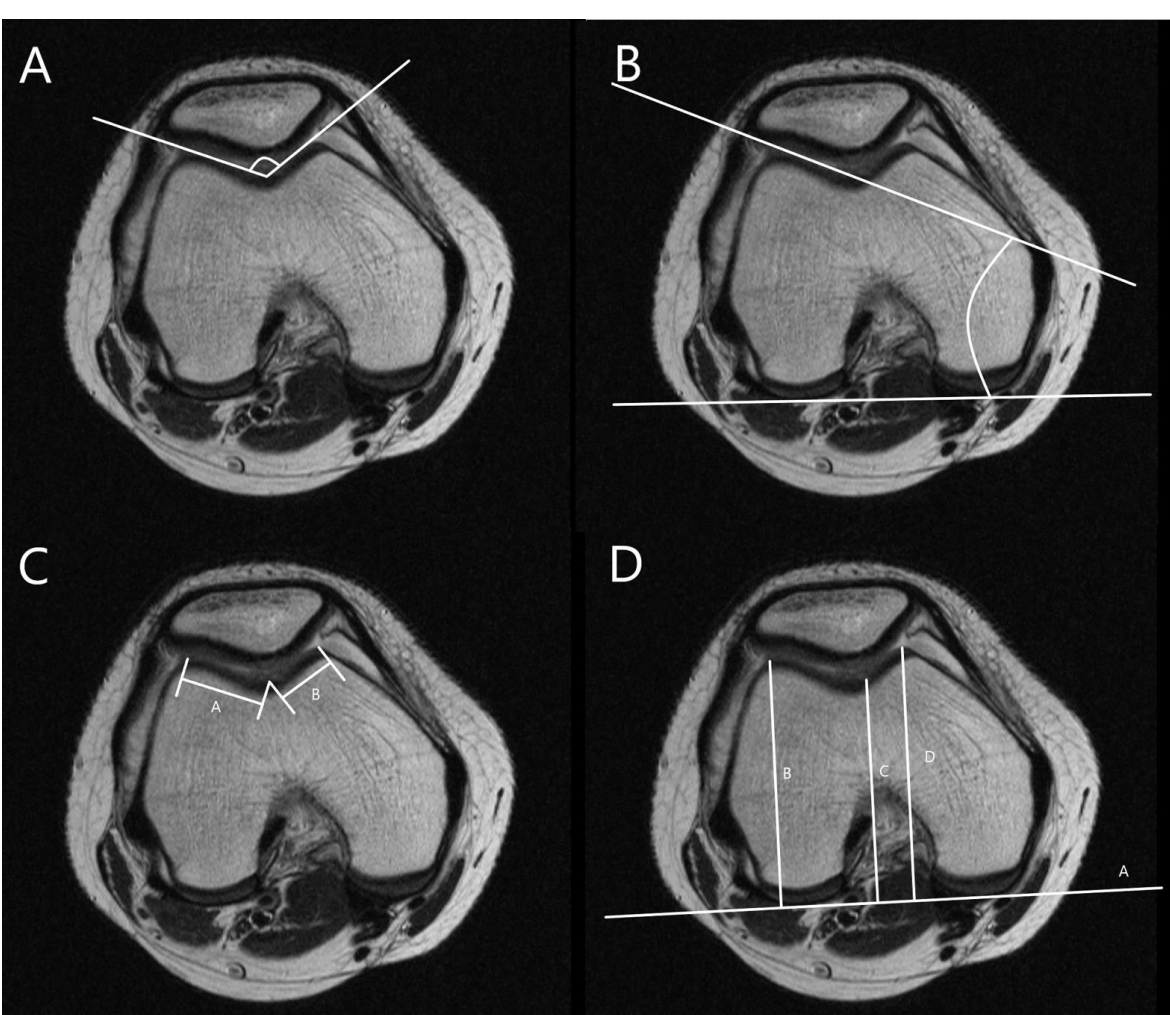
Measurements for trochlear morphology. (**A**) Measurement of sulcus angle (SA), the angle between slopes of the medial and lateral trochlea. (**B**) Measurement of lateral trochlear inclination (LTI), the angle between a line along with cartilage of lateral trochlear facet and a line along with posterior aspects of femoral condyles (posterior condylar axis). (**C**) Measurement of trochlear facet symmetry (TFS), the ratio of the length of medial trochlear facet (line B) to that of lateral trochlear facet (line A) [B/A]. (**D**) Measurement of femoral depth (FD), distance C between the deepest point of the trochlear sulcus and posterior condylar axis subtracted from a mean anteroposterior distance of medial and lateral femoral condyles (lines B and D) [B + D/2 − C].

The MRI was performed using a 1.5T (GE Healthcare, Milwaukee, WI, USA). The slice thickness was 3 mm in all sequences. The T1 fat saturation axial imaging was utilized to measure all parameters. The cartilaginous parameters of the patellofemoral morphology including sulcus angle (SA), lateral trochlear inclination (LTI), trochlear facet asymmetry (TFA), and femoral depth (FD) and alignment as well as tibial tuberosity–trochlear groove distance (TT-TG), percent sulcus location (PSL), and percent tibial tuberosity location (PTL) were assessed. Three orthopedic surgeons measured all parameters twice at 8-week intervals using image archiving and communication systems (Maroview, Marotech, Seoul, Korea). The morphological parameters of the patellofemoral joint were measured in the axial slice of MRI, which showed the deepest trochlear groove [18]. SA was defined as the angle between slopes of the medial and lateral trochlea [21,22]. LTI was measured as the angle between a line along with cartilage of lateral trochlear facet and the line along the posterior aspect of the femoral condyle (posterior condylar axis) [21]. TFA was defined as the ratio of the length of the medial trochlear facet to that of the lateral trochlear facet [21,23]. FD was defined as the distance between the deepest point of the trochlear sulcus and posterior condylar axis subtracted from the mean anteroposterior length of medial and lateral femoral condyles (Figure 2) [24]. 

**Figure 3:**
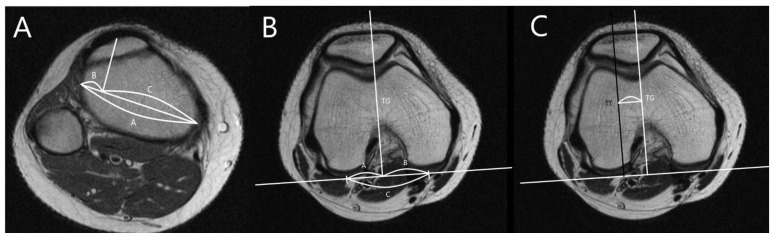
Position of the trochlear groove and tibial tuberosity. (**A**) Measurement of percent tibial tuberosity location (PTL). Lines B and C are divided by perpendicular lines from tibial tuberosity to line A [B/A × 100]. (**B**) Measurement of percent sulcus location (PSL). Lines E and F are divided by perpendicular lines from the deepest portion of the trochlear groove to a line along with posterior aspects of femoral condyles [E/D × 100]. (**C**) Measurement of tibial tuberosity—trochlear groove (TT-TG) distance, the distance from the deepest point of the trochlear groove to the tibial tuberosity.

The alignment parameters of the patellofemoral joint were measured in the axial slice of the deepest trochlear groove and the most prominent tibial tuberosity [25]. The PTL, PSL, and TT-TG were measured as shown in Figure 3 [26,27].

### 2.3. Statistical Analysis

The data were analyzed using PASW SPSS, version 22.0 (IBM Inc., Chicago, IL, USA). Descriptive statistics are presented as numbers and percentages according to age groups. The Kolmogorov–Smirnov test was used to assess normal distribution. ANOVA was performed to evaluate the significant difference between all age groups. The differences in parameters between age groups were evaluated via post hoc analysis. The results were reported as means, standard deviations, and 95% CI. *p* values < 0.05 were defined as significant. Intra- and interobserver reliability were evaluated by three surgeons based on the interclass correlation coefficient between measurements.

## 3. Results

A total of 663 MRI images (470 males, 193 females) were analyzed. The composition of the study population by age group is shown in Table 1. All measurements showed strong interobserver and intraobserver reliability. There were significant sex and age differences in TT-TG, SA, PTL, TFA, and FD (*p* < 0.05). The TT-TG showed the largest distance in the 13–15-year age group (male, 8.6 ± 3.8 mm; female, 7.2 ± 4.0 mm) and the smallest distance in the 4–6-year age group (male, 4.5 ± 2.8 mm; female, 4.1 ± 2.7 mm) for males and females. In males, the SA was the largest in the 7–9-year age group (146.3 ± 7.4°), while in females, it was the largest value in the 4–6-year age group (147.1 ± 7.7°). The PTL showed the largest value in the 4–6-year age group (33.8 ± 6.9 mm) and the smallest in the 10–12-year age group (26.6 ± 7.1 mm). The TFA showed generally similar values in both males and females. The FD increased steadily in males from 4–6 years to 16–18 years. In females, it increased and then decreased to the value reported in the 10–12-year age group (Table 2) (Tables S1–S6).

**Figure 4:**
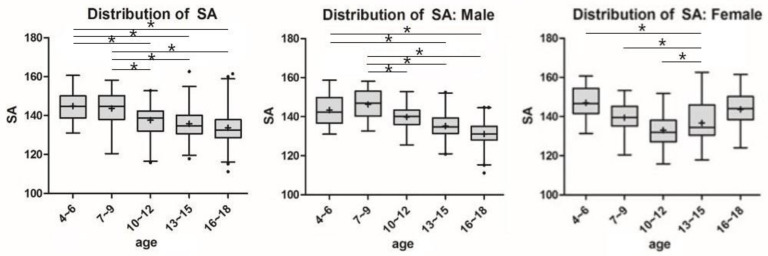
Parametric distribution of sulcus angle in MRI Measurements. The line and the asterisk indicate a significant difference between the two groups using post-hoc analysis. * means the *p* < 0.05.

**Figure 5:**
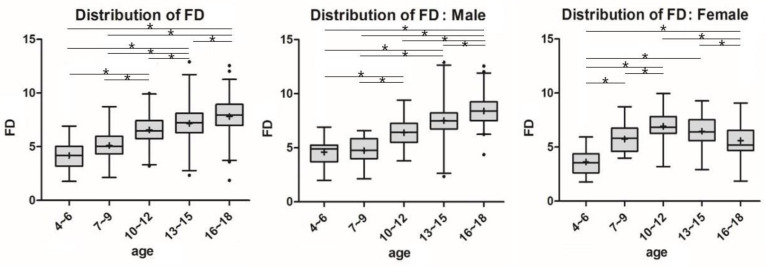
Parametric distribution of femoral depth (FD) in MRI Measurement. The line and the asterisk indicate a significant difference between the two groups using post-hoc analysis. * means the *p* < 0.05.

Significant differences in SA and FD between age groups for each sex are shown by lines and asterisks in Figure 4 and Figure 5. In males and females, SA and FD showed similar patterns in a reverse direction. Among males, the SA decreased from the age group of 7–9 years (146.3 ± 7.4°), and the FD increased with aging. However, in the females, the SA showed a pattern of decrease, followed by an increase in the age group of 10–12 years (133.0 ± 8.1°) as the inflection point, and the FD showed the opposite pattern (6.9 ± 1.4 mm).

## 4. Discussion

The main findings of the present study were the age-dependent differences in SA and FD patterns in males and females. In the male group, the SA showed a decline from the age group of 7–9 years, and the FD showed an increase with age. However, in females, the SA showed a pattern of decline, followed by an increase in the 10- to 12-year age group as the inflection point, and the FD showed the opposite trend. These results suggest that the sulcus groove was shallower after 10–12 years in females and support our primary hypothesis postulating sex-related developmental differences in the patellofemoral joint of the skeletally immature knee. 

Several previous studies reported that the incidence rate of acute primary patellar dislocation was the highest in women in their second decade of life [4,5,6,7]. In all sex groups, the highest risk for patellar dislocation among females was reported in the 10–17-year-old group [5]. A study based on the medical surveillance system of U.S. soldiers suggested that women were 61% more likely to experience patellar dislocation than men. Among them, groups under the age of 20 years carried an 84% higher risk than those above the age of 40 [6].

Femoral trochlear groove dysplasia was identified as one of the critical risk factors in patellar dislocation [5,28,29,30]. Trochlear dysplasia was reported in 96% of patients who underwent surgery for patellar dislocation compared with a mere 3% in those who did not [29]. Lewallen et al. reported that trochlear dysplasia and open physis were independent risk factors for recurrent patellar dislocation, while age, gender, BMI, and patellar alta were not [28]. In trochlear dysplasia, the trochlear groove is shallow in the proximal area, and concavity is less pronounced in the distal area. These anatomical properties result in significant loss of lateral patellar tracking, which results in lateral dislocation of the patella at the initiation of knee flexion. Additionally, due to the bilateral manifestations, it can be assumed that anomalies occur during development. 

Several studies compared anatomical geometry between normal and patellar dislocation groups. A prospective study of 103 skeletally immature children with a control group of 69 children reported that trochlear depth less than 3 mm was the key risk factor for anatomic patellar instability [16]. A matched-pair comparative study of 24 patients diagnosed with patellar instability suggested that patients with patellar instability had reduced trochlear volumes and depths compared with the unstable group [17].

Few studies investigated the development of normal skeletally immature knees. The results of two of the previous studies were inconsistent with the current study findings. The retrospective study of 246 knee MRIs reported SA measured at articular cartilage without age-related variation. Additionally, there were no age or sex differences in SA at articular cartilage. The average articular cartilage SA plateaued at 11 years in both sexes [18]. In the present study, female SA was less than 145° in the age group 7 years and older, but it increased significantly with aging. Another computed tomography scan of 31 skeletally immature fresh-frozen cadaveric knees reported a decrease in cartilaginous SA with age until after eight years and a plateau thereafter [21]. This study was conducted in a limited age group (2–11 years), and osseous measurements were made using CT. However, measurements involving articular cartilage may be more appropriate as they constitute the actual articular surface [31]. The retrospective analysis by Shital et al. showed no significant changes in cartilaginous and sulcus angles (average cartilaginous SA, 173.53°). Additionally, there were no significant differences between females and males. However, this study involved patients who underwent MPFL reconstruction. The foregoing studies appear to report similar results; however, these studies involved smaller sample sizes compared with our study. Second, racial differences need to be considered, given that both studies were conducted in western countries. Finally, comparisons of male and female SA differences were not clearly presented in both studies. Other studies involved normal trochlear morphology analysis, but this study design entailed a comparison between fetal and adult populations [32].

Our study will help in the understanding and diagnosis of patellar instability in children and adolescents. Clinically, in particular, in athletes with ha igh risk of injury, preventive rehabilitation training should be preceded by sex and age groups with a high probability of developing patella instability. [33,34] 

The study has a few limitations. First, as a large number of samples were included, the male-to-female ratio and the number of samples by age group differed in all age groups, but the difference was not statistically significant. Second, all MRIs were obtained with different knee flexion angles and variable cutoffs, so the actual axial sizes were determined along different axes. In addition, most of the images were 3 mm in size, and accurate images of the deepest trochlear groove could not be obtained. It would be better if the exact sequence and angle were set and determined by a radiologist. Third, the study population was divided by chronologic age rather than bone age. The accuracy of analysis of developmental changes can be increased by identifying bone age. Finally, the Q angle could not be analyzed because our study was performed retrospectively. Since Q angle is an important factor in determining patellofemoral instability, an age-correlated analysis of Q angle enables pathological evaluation.

## 5. Conclusions

Patellofemoral morphometry showed age and gender variations. Notably, the SA and FD varied significantly between males and females and differed according to the development. Females aged 10 to 12 years exhibit the deepest morphological features of the trochlear groove, which becomes shallower with maturity. These findings may reflect the sex differences and peak incidence of the patellar instability risk. Elucidation of the morphological changes and differences in the patellofemoral joint may facilitate the diagnosis of patellofemoral joint pathologies.

## Figures and Tables

**Table 1:** Composition of the study population by age group.

		Number (Percentage)	Total
**Age group (Male/Female)**	4–6 years	25 (3.8%)/18 (2.7%)	43 (6.5%)
	7–9 years	35 (5.3%)/21 (3.2%)	56 (8.4%)
	10–12 years	80 (12.1%)/38 (5.7%)	118 (17.8%)
	13–15 years	117 (17.6%)/59 (8.9%)	176 (26.5%)
	16–18 years	213 (32.1%)/57 (8.6%)	270 (40.7%)
	Total	470 (70.9%)/193 (29.1%)	663 (100%)

**Table 2:** Patellofemoral Morphological Measurements.

	Age Group (Mean ± SD and 95% Confidence Interval)				
	1	2	3	4	5
**Overall**					
**TT-TG distance (mm)**	4.3 ± 2.7 (3.49–5.11)	5.5 ± 3.1 (4.69–6.31)	5.9 ± 3.2 (5.32–6.48)	7.9 ± 3.9 (7.32–8.48)	7.1 ± 4.3 (6.59–7.61)
**Sulcus angle (degree)**	144.9 ± 7.9 (143–147)	143.7 ± 8.2 (142–146)	137.7 ± 7.6 (136–139)	136.2 ± 9.9 (135–138)	133.2 ± 10.5 (132–134)
**Percent sulcus location (%)**	43.9 ± 6.6 (41.9–45.9)	44.8 ± 6.6 (43.1–46.5)	42.2 ± 5.0 (41.3–43.1)	42.3 ± 5.5 (41.5–43.1)	43.8 ± 10.5 (42.5–45)
**Percent Tibia tuberosity location (%)**	33.8 ± 6.9 (31.7–35.9)	30.4 ± 6.4 (28.7–32.1)	26.6 ± 7.1 (25.3–27.9)	28.3 ± 6.0 (27.4–29.2)	30.4 ± 4.8 (29.8–31)
**Lateral trochlear inclination (degree)**	20.8 ± 6.3 (18.9–22.7)	21.2 ± 4.8 (19.9–22.5)	21.2 ± 4.5 (20.4–22)	21.8 ± 5.4 (21–22.6)	22.5 ± 5.1 (21.9–23.1)
**Trochlear facet symmetry (ratio)**	0.9 ± 0.3 (0.81–0.99)	0.8 ± 0.3 (0.72–0.88)	0.8 ± 0.1 (0.782–0.818)	0.7 ± 0.1 (0.685–0.715)	0.7 ± 0.1 (0.69–0.71)
**Femoral depth (mm)**	4.0 ± 1.8 (3.46–4.54)	4.9 ± 1.9 (4.4–5.4)	6.6 ± 1.3 (6.36–6.83)	7.0 ± 2.2 (6.67–7.33)	8.0 ± 3.6 (7.57–8.43)
***Male***					
**TT-TG distance (mm)**	4.5 ± 2.8 (3.4–5.6)	6.2 ± 3.0 (5.21–7.19)	6.4 ± 2.8 (5.79–7.01)	8.3 ± 3.8 (7.61–8.99)	7.4 ± 4.3 (6.82–7.98)
**Sulcus angle (degree)**	143.4 ± 7.9 (140–147)	146.3 ± 7.4 (144–149)	140.0 ± 6.4 (139–141)	136.0 ± 10.0 (134–138)	130.4 ± 9.1 (129–132)
**Percent sulcus location (%)**	42.8 ± 6.1 (40.4–45.2)	44.0 ± 5.8 (42.1–45.9)	42.4 ± 4.2 (41.5–43.3)	42.0 ± 4.3 (41.2–42.8)	43.4 ± 9.7 (42.1–44.7)
**Percent Tibia tuberosity location (%)**	32.4 ± 6.5 (29.8–34.9)	29.0 ± 6.3 (26.9–31.1)	24.5 ± 6.4 (23.1–25.9)	26.6 ± 5.3 (25.6–27.6)	29.8 ± 4.3 (29.2–30.4)
**Lateral trochlear inclination (degree)**	20.6 ± 7.2 (17.8–23.4)	20.1 ± 4.5 (18.6–21.6)	20.1 ± 3.8 (19.3–20.9)	21.6 ± 5.8 (20.6–22.7)	22.1 ± 4.3 (21.5–22.7)
**Trochlear facet symmetry (ratio)**	0.9 ± 0.4 (0.743–1.06)	0.9 ± 0.4 (0.767–1.03)	0.8 ± 0.1 (0.778–0.822)	0.7 ± 0.1 (0.682–0.718)	0.7 ± 0.1 (0.687–0.713)
**Femoral depth (mm)**	4.2 ± 2.2 (3.34–5.06)	4.5 ± 2.0 (3.84–5.16)	6.4 ± 1.2 (6.14–6.66)	7.3 ± 2.5 (6.85–7.75)	8.7 ± 3.7 (8.2–9.2)
***Female***					
**TT-TG distance (mm)**	4.1 ± 2.7 (2.85–5.35)	4.3 ± 3.0 (3.02–5.58)	4.9 ± 3.6 (3.76–6.04)	7.2 ± 4.0 (6.18–8.22)	6.3 ± 3.9 (5.29–7.31)
**Sulcus angle (degree)**	147.1 ± 7.7 (144–151)	139.5 ± 8.0 (136–143)	133.0 ± 8.1 (130–136)	136.7 ± 9.5 (134–139)	143.7 ± 8.6 (141–146)
**Percent sulcus location (%)**	45.4 ± 7.0 (42.2–48.6)	46.1 ± 7.7 (42.8–49.4)	41.8 ± 6.2 (39.8–43.8)	42.9 ± 7.4 (41–44.8)	45.1 ± 13.0 (41.7–48.5)
**Percent Tibia tuberosity location (%)**	35.5 ± 7.2 (32.2–38.8)	33.0 ± 5.9 (30.5–35.5)	30.7 ± 6.7 (28.6–32.8)	31.3 ± 6.6 (29.6–33)	32.7 ± 5.6 (31.3–34.2)
**Lateral trochlear inclination (degree)**	21.2 ± 4.9 (18.9–23.5)	22.9 ± 4.8 (20.8–24.9)	23.8 ± 5.0 (22.2–25.4)	22.0 ± 4.4 (20.9–23.1)	23.9 ± 7.3 (22–25.8)
**Trochlear facet symmetry (ratio)**	0.9 ± 0.2 (0.808–0.992)	0.7 ± 0.2 (0.614–0.785)	0.8 ± 0.1 (0.768–0.832)	0.7 ± 0.2 (0.649–0.751)	0.7 ± 0.3 (0.622–0.778)
**Femoral depth (mm)**	3.6 ± 1.2 (3.05–4.15)	5.7 ± 1.3 (5.14–6.26)	6.9 ± 1.4 (6.46–7.35)	6.5 ± 1.4 (6.14–6.86)	5.5 ± 1.6 (5.08–5.92)

## Data Availability

Not applicable.

## Supplementary Materials

The following are available online at https://www.mdpi.com/article/10.3390/diagnostics11111985/s1, Table S1: Posthoc tukey test (Sulcus angle)-All sex, Table S2: Posthoc tukey test (Sulcus angle)-Male, Table S3: Posthoc tukey test (Sulcus angle)-Female, Table S4: Posthoc tukey test (Femoral depth)-All sex, Table S5: Posthoc tukey test (Femoral depth)-Male, Table S6: Posthoc tukey test (Femoral depth)-Female.
