# Supplementary material for: Magnetic Resonance Imaging of Patellofemoral Morphometry Reveals Age and Gender Variations in the Knees of Children and Adolescents
Source: Diagnostics (Basel). 2021 Oct 26;11(11):1985. doi: 10.3390/diagnostics11111985 (PMC8618599; doi:10.3390/diagnostics11111985)
Supplement: Supplementary file 1 [file diagnostics-11-01985-s001.zip › diagnostics-1421080-supplementary.pdf]

Supplementary Table S1. Posthoc tukey test (Sulcus angle)-All sex

Comparisons significant at the 0.05 level are indicated by *.

Age group Comparison	Difference Between Means	Simultaneous 95% Confidence Limits		
4 vs 5	2.8449	-4.2519	9.9417	
4 vs 3	4.0503	-2.762	10.8626	
4 vs 2	10.1114	3.8241	16.3987	*
4 vs 1	11.5335	5.4112	17.6559	*
5 vs 4	-2.8449	-9.9417	4.2519	
5 vs 3	1.2054	-4.2842	6.695	
5 vs 2	7.2665	2.4438	12.0892	*
5 vs 1	8.6886	4.083	13.2942	*
3 vs 4	-4.0503	-10.8626	2.762	
3 vs 5	-1.2054	-6.695	4.2842	
3 vs 2	6.0611	1.6678	10.4544	*
3 vs 1	7.4832	3.3294	11.637	*
2 vs 4	-10.1114	-16.3987	-3.8241	*
2 vs 5	-7.2665	-12.0892	-2.4438	*
2 vs 3	-6.0611	-10.4544	-1.6678	*
2 vs 1	1.4221	-1.7992	4.6434	
1 vs 4	-11.5335	-17.6559	-5.4112	*
1 vs 5	-8.6886	-13.2942	-4.083	*
1 vs 3	-7.4832	-11.637	-3.3294	*
1 vs 2	-1.4221	-4.6434	1.7992	

Supplementary Table S2. Posthoc tukey test (Sulcus angle)-Male

Comparisons significant at the 0.05 level are indicated by *.

Age group Comparison	Difference Between Means	Simultaneous 95% Confidence Limits		
2 vs 1	2.8846	-3.6523	9.4214	
2 vs 3	6.4248	1.3658	11.4839	*
2 vs 4	10.2711	5.4617	15.0806	*
2 vs 5	15.8823	11.3293	20.4354	*
1 vs 2	-2.8846	-9.4214	3.6523	
1 vs 3	3.5403	-2.1795	9.26	
1 vs 4	7.3866	1.8863	12.8868	*
1 vs 5	12.9978	7.7203	18.2752	*
3 vs 2	-6.4248	-11.4839	-1.3658	*
3 vs 1	-3.5403	-9.26	2.1795	
3 vs 4	3.8463	0.2248	7.4679	*
3 vs 5	9.4575	6.1841	12.7309	*
4 vs 2	-10.2711	-15.0806	-5.4617	*
4 vs 1	-7.3866	-12.8868	-1.8863	*
4 vs 3	-3.8463	-7.4679	-0.2248	*
4 vs 5	5.6112	2.7386	8.4838	*
5 vs 2	-15.8823	-20.4354	-11.3293	*
5 vs 1	-12.9978	-18.2752	-7.7203	*
5 vs 3	-9.4575	-12.7309	-6.1841	*
5 vs 4	-5.6112	-8.4838	-2.7386	*

Supplementary Table S3. Posthoc tukey test (Sulcus angle)-Female

Comparisons significant at the 0.05 level are indicated by *.

Age group Comparison	Difference Between Means	Simultaneous 95% Confidence Limits		
1 vs 5	3.393	-3.302	10.087	
1 vs 2	7.594	-0.359	15.548	
1 vs 4	10.356	3.689	17.023	*
1 vs 3	14.008	6.923	21.093	*
5 vs 1	-3.393	-10.087	3.302	
5 vs 2	4.202	-2.119	10.523	
5 vs 4	6.963	2.365	11.562	*
5 vs 3	10.616	5.43	15.801	*
2 vs 1	-7.594	-15.548	0.359	
2 vs 5	-4.202	-10.523	2.119	
2 vs 4	2.762	-3.53	9.054	
2 vs 3	6.414	-0.319	13.147	
4 vs 1	-10.356	-17.023	-3.689	*
4 vs 5	-6.963	-11.562	-2.365	*
4 vs 2	-2.762	-9.054	3.53	
4 vs 3	3.652	-1.498	8.803	
3 vs 1	-14.008	-21.093	-6.923	*
3 vs 5	-10.616	-15.801	-5.43	*
3 vs 2	-6.414	-13.147	0.319	
3 vs 4	-3.652	-8.803	1.498	

Supplementary Table S4. Posthoc tukey test (Femoral depth)-All sex

Comparisons significant at the 0.05 level
are indicated by *.

Age group Comparison	Difference Between Means	Simultaneous 95% Confidence Limits		
4 vs 5	0.9505	0.208	1.6931	*
4 vs 3	1.4262	0.5804	2.272	*
4 vs 2	3.0534	1.928	4.1789	*
4 vs 1	4.0058	2.7474	5.2643	*
5 vs 4	-0.9505	-1.6931	-0.208	*
5 vs 3	0.4756	-0.4363	1.3876	
5 vs 2	2.1029	0.927	3.2788	*
5 vs 1	3.0553	1.7515	4.3591	*
3 vs 4	-1.4262	-2.272	-0.5804	*
3 vs 5	-0.4756	-1.3876	0.4363	
3 vs 2	1.6273	0.3835	2.871	*
3 vs 1	2.5796	1.2144	3.9449	*
2 vs 4	-3.0534	-4.1789	-1.928	*
2 vs 5	-2.1029	-3.2788	-0.927	*
2 vs 3	-1.6273	-2.871	-0.3835	*
2 vs 1	0.9524	-0.6017	2.5065	
1 vs 4	-4.0058	-5.2643	-2.7474	*
1 vs 5	-3.0553	-4.3591	-1.7515	*
1 vs 3	-2.5796	-3.9449	-1.2144	*
1 vs 2	-0.9524	-2.5065	0.6017	

Supplementary Table S5. Posthoc tukey test (Femoral depth)-Male

Comparisons significant at the 0.05 level are indicated by *.

Age group Comparison	Difference Between Means	Simultaneous 95% Confidence Limits		
2 vs 1	2.8846	-3.6523	9.4214	
2 vs 3	6.4248	1.3658	11.4839	*
2 vs 4	10.2711	5.4617	15.0806	*
2 vs 5	15.8823	11.3293	20.4354	*
1 vs 2	-2.8846	-9.4214	3.6523	
1 vs 3	3.5403	-2.1795	9.26	
1 vs 4	7.3866	1.8863	12.8868	*
1 vs 5	12.9978	7.7203	18.2752	*
3 vs 2	-6.4248	-11.4839	-1.3658	*
3 vs 1	-3.5403	-9.26	2.1795	
3 vs 4	3.8463	0.2248	7.4679	*
3 vs 5	9.4575	6.1841	12.7309	*
4 vs 2	-10.2711	-15.0806	-5.4617	*
4 vs 1	-7.3866	-12.8868	-1.8863	*
4 vs 3	-3.8463	-7.4679	-0.2248	*
4 vs 5	5.6112	2.7386	8.4838	*
5 vs 2	-15.8823	-20.4354	-11.3293	*
5 vs 1	-12.9978	-18.2752	-7.7203	*
5 vs 3	-9.4575	-12.7309	-6.1841	*
5 vs 4	-5.6112	-8.4838	-2.7386	*

Supplementary Table S6. Posthoc tukey test (Femoral depth)-Female

Comparisons significant at the 0.05 level
are indicated by *.

Age group Comparison	Difference Between Means	Simultaneous 95% Confidence Limits		
3 vs 4	0.4583	-0.3815	1.2981	
3 vs 2	1.1892	0.0914	2.2871	*
3 vs 5	1.416	0.5704	2.2616	*
3 vs 1	3.2954	2.1401	4.4507	*
4 vs 3	-0.4583	-1.2981	0.3815	
4 vs 2	0.731	-0.295	1.7569	
4 vs 5	0.9577	0.2078	1.7076	*
4 vs 1	2.8371	1.7499	3.9243	*
2 vs 3	-1.1892	-2.2871	-0.0914	*
2 vs 4	-0.731	-1.7569	0.295	
2 vs 5	0.2267	-0.804	1.2574	
2 vs 1	2.1062	0.8093	3.4031	*
5 vs 3	-1.416	-2.2616	-0.5704	*
5 vs 4	-0.9577	-1.7076	-0.2078	*
5 vs 2	-0.2267	-1.2574	0.804	
5 vs 1	1.8795	0.7878	2.9711	*
1 vs 3	-3.2954	-4.4507	-2.1401	*
1 vs 4	-2.8371	-3.9243	-1.7499	*
1 vs 2	-2.1062	-3.4031	-0.8093	*
1 vs 5	-1.8795	-2.9711	-0.7878	*
